# Preliminary transcriptomic analyses reveal *in vitro* and *in planta* overexpression of various bacteriocins in *Xylella fastidiosa*

**DOI:** 10.3389/fmicb.2025.1501741

**Published:** 2025-02-21

**Authors:** Serafina Serena Amoia, Maria Saponari, Pasquale Saldarelli, Angela Maria Ligorio, Carmine Del Grosso, Giuliana Loconsole, Giusy D’Attoma, Donato Boscia, Annalisa Giampetruzzi

**Affiliations:** ^1^Institute for Sustainable Plant Protection, National Research Council (IPSP-CNR), Bari, Italy; ^2^Department of Soil, Plant and Food Sciences, University of Bari Aldo Moro, Bari, Italy

**Keywords:** transcriptomics, dual RNA-seq, host-pathogen interaction, bacteriocin, replication biomarker, early detection, *Xylella fastidiosa*

## Abstract

**Introduction:**

*Xylella fastidiosa* is a phytopathogenic bacterium of worldwide importance causing detrimental diseases in several crops. Recent reports from European and Mediterranean countries raised great concerns and have given impetus to new studies investigating both the pathogenicity of the newly emerged strains and the susceptibility and vulnerability of Mediterranean agroecosystems, with the outbreak in olive trees in southern Italy being the most investigated new pathosystem. The complexity of this pathogen makes difficult to understand its interaction mechanisms with host plants and plant microbial communities.

**Materials and methods:**

In this study, we performed a pilot dual RNA-seq analysis on a diseased olive tree infected by *Xylella fastidiosa* subspecies *pauca*, to gather information about bacterial infection dynamics and reciprocal interactions between plant host and the bacterium. Adopting a mRNA enrichment protocol allowed to better probe bacterial sequences by increasing the resolution of differential gene expressions.

**Results:**

The overexpression of a bacteriocin (*cvaC-1*), as the major result gained by the transcriptomic analysis, led us to validate its potential application as a marker of *Xylella fastidiosa* multiplication in olive, citrus and periwinkle artificially inoculated plants. Transcriptomic analysis of *in vitro* cultured strains of *Xylella fastidiosa* subspecies *pauca*, while confirming that bacteriocin-related genes are the most abundant transcripts, unraveled strain differences in the *cvaC-1* and *cvaC-2* ratio.

**Discussion:**

Our findings suggest that the *cvaC-1*-related transcript can be employed in RT-qPCR/RT-PCR to improve the detectability of actively growing *Xylella fastidiosa* cells *in vitro* and in host’s xylem vessels. Indeed, being the most expressed component of bacterial weapons, novel studies focusing on its functions and role in the bacterial pathogenic life cycle should be envisioned.

## 1 Introduction

*Xylella fastidiosa* (*Xf*), *Lysobacteraceae* family, *Gammaproteobacteria* class (Wells et al., 1987) is a fastidious, non-flagellate, Gram-negative, xylem-limited bacterium, known to cause destructive diseases worldwide in a broad range of crops (grapevine, citrus, stone fruit, olive, pecan, blueberry, alfa alfa, and coffee) but also ornamental and forestry plant species (elm, oak, sycamore, and oleander) (Henneberger et al., 2004; Rapicavoli et al., 2018). The updated *Xf* host plant database lists over 712 species across 312 genera and 89 families in which the bacterium causes symptomatic or latent infections (Cavalieri et al., 2024). The *Xf* long-distance spread and its associated diseases are primarily attributed to human activities related to the movement and exchange of infected plant material while locally, the bacterium is dispersed through xylem-sap feeding insects (Almeida and Nunney, 2015). *Xf* was originally discovered in the late 1800’s in the Americas, where is now endemically distributed and more recently, in Asia and Europe, being reported, up to now, in more than 20 countries across America, Asia, and Europe [PM 7/24 (5) Xylella fastidiosa, 2023]. The first *Xf* finding in Europe occurred in 2013, associated with the Olive Quick Decline Syndrome (OQDS) (Saponari et al., 2013, 2014; Cariddi et al., 2014), a severe epidemic decimating olive trees in the Salento peninsula (Apulia, Italy), although phylogenetic and genomic investigations dated the first introduction of the pathogen in the European continent back to the 80’s of the last century (Soubeyrand et al., 2018; Landa et al., 2020).

The plant-bacterial interactions enhancing systemic host colonization and modulating plant defense response are still largely unknown. It is well-known that *Xf* has a dual lifestyle encompassing a planktonic motile form to systemically colonize the plant vessels, and a steady condition, where cells are sticky and organized in aggregates embedded in an exopolysaccharide (EPS) matrix, which is required by the insect vector for the acquisition and transmission (Chatterjee et al., 2008), passing through a phase transition that is finely regulated by a mechanism called “quorum sensing.”

**FIGURE 1:**
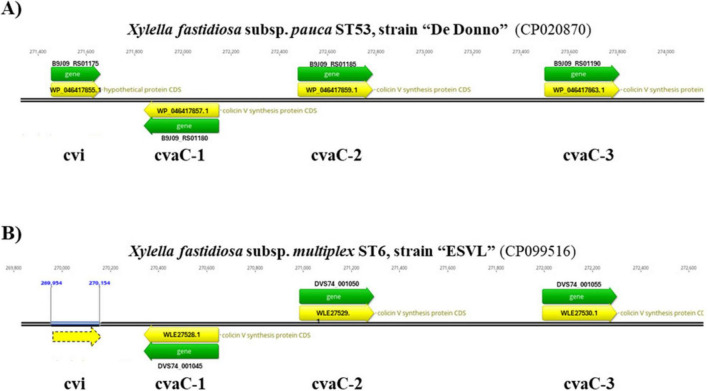
Tripled-tandem organization of the *cvaC* operon. The correspondent genome regions in the *Xfp* De Donno (Acc. Nr. CP020870.1) **(A)** and *Xfm* ESVL (Acc. Nr. CP099516.1) genome **(B)** are displayed. In both genomes*, cvaC-1* gene is in antisense direction and the self-immunity protein, encoded by *cvi* gene, is placed upstream of the *cvaC* operon. In the ESVL genome, the gene coding for the *cvi* protein is not annotated, even if an ORF (dashed yellow arrow) is present in the sequence. Gene ID (Locus tag) and Protein IDs are also reported.

A further process regulating the cell growth dynamics, the bacterium survival to various stresses, and the virulence is based on bacterial toxin-antitoxin (TA) systems (Merfa et al., 2016; de Souza-Neto et al., 2022). TA loci are organized in operons and encode for two neighboring genes, a stable protein-based “toxin,” that could exert harmful effects on the cells inhibiting their growth, and a specific cognate labile “antitoxin” (either RNA or protein), that acts as a transcriptional repressor, preventing self-toxicity (Maisonneuve and Gerdes, 2014). This auto-inhibition, called “conditional cooperativity,” relies on the proper toxin: antitoxin stoichiometric ratio in the cells under normal conditions (Cataudella et al., 2012), while the uneven co-expression of TA genes have been associated with cell growth dynamics under stress conditions (Burbank and Stenger, 2017; Gupta et al., 2017). *Xf* has multiple TA modules on its chromosome involved both in virulence and stress survival (Van Sluys et al., 2003; Lee et al., 2014). One such TA system, named *cvaC*/*cvi*, represents a putative component of the virulence process (Zaini et al., 2008). The *cvaC* toxin belongs to the bacteriocin class, sharing homologies with the RTX protein recovered in *Rizobium leguminosarum*, a hemolysin and leukotoxin (Oresnik et al., 1999; Holtsmark et al., 2008) that possibly plays a structural role in biofilm maturation (de Souza et al., 2020). *Xf* bacteriocin has a comparable structure with colicin V (Col-V) secreted by *E. coli* and other *Enterobacteriaceae* members (Waters and Crosa, 1991). The peptide, synthesized as a 103 amino acids precursor molecule, endowed with a leader 15-amino acid motif, is successively post-translationally modified during its secretion by the proteolytic cleavage of the leader to give rise to the 88 amino acid long mature protein. This “core peptide” represents the bioactive molecule. Indeed, the abundant levels of transcript of this gene, under standard growth conditions (De Souza et al., 2003) and in microbiological media supplemented with glucose (Pashalidis et al., 2005), iron (Zaini et al., 2008), calcium (Chen and De La Fuente, 2020) and grape sap (Nunes et al., 2010; Surano et al., 2023) confirms that the toxin encoded by *cvaC* gene is likely a prevalent weapon employed by *Xf* to efficiently compete with other closely-related sensitive endophytes in the xylem and insect foregut (Zaini et al., 2008). In most of *Xf* genomes publicly available, *cvaC* gene has a tripled-tandem organization, with the first copy (*cvaC-1*) in antisense and the other two (*cvaC-2* and *cvaC-3*) in sense direction. Located upstream of this module is the immunity protein *cvi*, which self-protects the producing strain from the toxic effects of its own antibacterial product (Pashalidis et al., 2005; Baquero et al., 2019; Figure 1). Because of the molecular mass of *cvaC* below 10 kDa, and the existence of a dedicated export system for the translocation, it should fall in the microcin group (MccV-like) (Duquesne et al., 2007) likely acting as a pore-forming toxin (PFT), causing membrane permeabilization and destabilization, i.e., the formation of pores and channels in the lipid bilayers, through the binding of specific receptors, and therefore, the dissipation of the protonmotive force of sensitive bacteria (Chatterjee et al., 2005; Hasper et al., 2006).

While several transcriptome studies provided insights into the plant response to *Xf* infection (Rodrigues et al., 2013; Giampetruzzi et al., 2016; Zaini et al., 2018), the bacterial gene expression in infected plants has been poorly investigated (Surano et al., 2023) or limited to the condition mimicking the xylem environment (De Souza et al., 2003; Parker et al., 2016; Chen and De La Fuente, 2020; Wei et al., 2021). The application of dual RNA sequencing (dRNA-seq) allows for the simultaneous study of the host and the pathogen transcriptomes, detecting pathogen-specific transcripts and host responses in the same tissues, thus providing a comprehensive understanding of their interaction (Westermann et al., 2012) and revealing novel aspects of the infective process. Besides shedding light on the *Xf*/host interaction, such studies could identify genes of the pathogen to exploit as biomarkers activated at the early stage of the infection, allowing to develop new molecular approaches based on the replication activity and physiological/viability state of the microorganism in a sample (Kovalchuk et al., 2019; Kunjeti et al., 2012; Meyer et al., 2016). Indeed, the identification of biomarkers linked to the primary phase of the infection, prior the symptoms onset and before the pathogen population reaches detectable level, is crucial for successful early detection.

In the present work, a pilot dRNA-seq experiment was carried out to profile the *Xf* subsp. *pauca* (*Xfp*) gene expression in a naturally infected olive tree and the data, compared to the transcriptome profile of *in vitro* grown *Xf*, allowed us to identify a bacterial transcript highly expressed *in planta* during the infection process and conserved across strains belonging to different subspecies. The transcript, annotated as a “Colicin V synthesis protein” (*cvaC-1*) coding for a virulence-related microcin, resulted in an effective molecular target for a timely diagnosis of *Xf* active growing cells in tissues of different hosts, by quantitative reverse transcription PCR (RT-qPCR) or conventional two-step RT-PCR.

The developed assay proved to be more sensitive (with higher analytical sensitivity) than the official real-time quantitative PCR (qPCR, Harper et al., 2010) test widely used for bacterial detection. Indeed, when the results of the RT-qPCR assay were compared with those of the standard qPCR targeting the bacterial DNA, it was possible to retrieve additional useful indications about the *status* of the infection in the host plants, i.e., active replicating and colonizing the plant *vs* non-growing bacterial cells.

## 2 Materials and methods

### 2.1 Bacterial strains and growth conditions

*Xf* strains used in our experiments belonged to subspecies *pauca* and *multiplex* (*Xfm*). More specifically, *Xfp* strains “De Donno” (later on DD) (CFBP 8402), APL-64 and APL-69, all belonging to sequence type (ST) 53, were isolated between 2013 and 2017 from OQDS affected olive trees in the Salento area (Apulia, Italy) (Giampetruzzi et al., 2017a; Saponari et al., 2017; Sicard et al., 2021). *Xfm* strain ESVL (CFBP 8684) ST6 was recovered from an almond tree in Guadalest Valley (Alicante, Spain) with notable almond leaf scorch disease signs (ALSD) (Giampetruzzi et al., 2019b). Moreover, *Xfm* strain “TOS4” having ST87 genotype, was recovered from an infected almond in the Italian region of Tuscany (northern Italy) (Giampetruzzi et al., 2019a) All strains were cultured for 10 days on PD3 agar plates (Davis, 1992) at 28°C from −80°C glycerol stock (50%), re-streaked onto new PD3 plates and cultured for another 7 days before use.

### 2.2 Total DNA extraction and detection of *Xylella fastidiosa* in infected plants

Tissues for the dual RNA-seq were sampled from a *Xfp*-infected olive of the susceptible cultivar Ogliarola salentina grown in the Salento epidemic area. Wooden shoots were collected from the sections of the canopy close to branches with severe symptoms of desiccation of 1 or 2 years-old twigs (*ca*. 0.5 cm in diameter and 1–1.5 cm long) were debarked with a scalpel to obtain *ca*. 1 g of xylem-enriched tissues that was used to extract total DNAs and RNAs fractions. Total DNAs were extracted using the PureFood GMO and Authentication Kit on the dedicated automatic platform Maxwell^®^ RSC (Promega Corporation, United States) (Loconsole et al., 2021) and processed to assess the *Xf* presence using a qPCR assay following the protocol of (Harper et al., 2010), with some minor modifications (Amoia et al., 2023).

### 2.3 Construction of dual RNA-seq library from infected olive tree

Total RNAs extraction was carried out on xylem-enriched tissues of olive obtained as described above. One *g* of twigs was grinded in a mortar using 10 mL of cold McKenzie grinding buffer (Foissac et al., 2001) plus 2% sodium metabisulfite. The homogenate was added with 20% N-Lauroylsarcosine Sodium salt and centrifuged (13.000 × *g*) to throw out the plant debris. The clear slurry was extracted twice with TRIzol™ (Invitrogen™) and chloroform in a proportion of 1:1 (*v:v*) by 5.000 ×* g* centrifugation, and the final supernatant was added with 2.5 volumes of 70% ethanol and stored overnight at −20°C. Total RNAs were recovered by centrifugation at 20.000 × *g* and resuspended in 450 μL RTL buffer (RNeasy Plant Mini Kit, Qiagen) to be further purified according to the manufacturer’s instructions. RNA concentration and quality were evaluated by measuring the absorbance ratio 260/280 nm with the spectrophotometer NanoPhotometer™ N60 UV/Vis (Implen, München Germany). RNA quality was assessed by evaluating the integrity of 28S and 18S rRNA bands on 1.2% TBE (Tris boric EDTA) agarose gel and by capillary electrophoresis (Supplementary Figure 1).

Total RNAs were used for the dRNA-seq library construction which was outsourced to Vertis Biotechnology AG, Germany^1^, according to a custom protocol based on depletion of ribosomal RNA (rRNA) molecules (plant plus bacteria). The ribodepleted RNAs were first fragmented by ultrasounds (2 pulses of 30 s each at 4°C). Then, an oligonucleotide adapter was ligated to the 3′ end of the fragmented RNA molecules. First-strand cDNA synthesis was primed by an oligonucleotide complementary to the 3′ adapter using M-MLV reverse transcriptase. The first strand cDNA was purified, and the 5’ Illumina TruSeq sequencing adapter was ligated to the 3′ end of the antisense cDNA. The resulting cDNA was PCR-amplified (16 cycles) to achieve about 10–20 ng/μL of DNA using a high-fidelity DNA polymerase and the TruSeq barcode sequences. The cDNA was purified using the Agencourt AMPure XP kit (Beckman Coulter Genomics) and analyzed by capillary electrophoresis (Supplementary Figure 3). For the Illumina NextSeq sequencing, the DNA sample was size-fractionated in the range of 200–550 bp using a preparative agarose gel. An aliquot was also analyzed by capillary electrophoresis (Supplementary Figure 3). The DNA pool was sequenced on an Illumina NextSeq 500 system using 1 × 75 bp read length.

### 2.4 RNA-seq on *in vitro* bacterial cultures

Three different *Xfp* ST53 strains (Xfp DD, APL-64, APL-69) grown *in vitro* were used for RNASeq analysis. Upon scraping bacterial colonies and dissolving them in sterile water to obtain a turbid suspension, cells were centrifugated at 10.000 × *g* for 10 min at 4°C. Total RNAs were extracted from the three bacterial pellets according to the TRIzol™ Reagent protocol (Invitrogen™) and successively DNAse digested with RNase-free DNase I following the manufacturer’s instruction (Thermo Scientific™), to avoid contamination by genomic DNA. Purity, concentration and integrity of RNA samples were evaluated by agarose gel electrophoresis as described above. RNA samples with a RNA:DNA ratio > 15 and RNA integrity number (RIN) > 7 were employed for libraries construction. Libraries were prepared with the TruSeq stranded total RNA kit construction for microbe with NEBNext^®^ rRNA Depletion Kit (Bacteria). Paired-end sequencing (100 × 2 bp) was performed on an Illumina Novaseq 6000 platform at the Macrogen Europe B.V. facilities (Amsterdam, NE).

### 2.5 Bioinformatic pipeline analysis of the RNASeq and dRNA-seq libraries

The FastQC tool software^2^ was used to assess the quality of the raw reads obtained by the two different RNASeq sequencing approaches. The reference genome data of *Olea europaea* L. subsp. *europaea* var. *europaea* cv Farga v9 (Julca et al., 2020) and *Xfp* DD (Giampetruzzi et al., 2017b) were downloaded from NCBI database^3^. Raw reads obtained from the dRNA-seq, preliminarily checked for quality and trimmed of the adapters, were first mapped to the cv. Farga genome v9 using TopHat2 (Kim et al., 2013). The remaining unmapped reads were then mapped to the *Xfp* DD genome. In parallel, raw reads obtained from the RNASeq of the *in vitro* cultured bacteria were directly mapped to the *Xfp* DD bacterial genome. Reads count data after mapping on the reference genome (*Xfp* DD bacterial genome) were determined using SeqMonk software version 1.48.1^4^. Gene expression levels of both RNASeq and dRNASeq were measured as raw count reads *per* annotated transcript by SeqMonk. Therefore, two abundance matrices of reads to the annotated bacterial genome were obtained and reported in the Excel file for further comparison between the two. The number of counts *per* annotated transcript was also normalized to 20,000, which was the lowest amount of total mapped reads obtained from the dRNA-seq library.

### 2.6 RT-PCR and RT-qPCR for gene expression and *Xf* transcripts detection

#### 2.6.1 Gene expression analysis for validation of the RNA-seq results

A Sybr-green-based two-step RT-qPCR assay was developed to target three out of four genes forming the *cvaC* operon in *Xf* genome. Primers for *cvaC-1*, *cvaC-2* and *cvi* genes were designed using Primer-Blast tool version 4.1.0, available at NCBI website, and checked *in silico* for their specificity to target transcripts. The tandem arrangement of the three *cvaC* copies and the putative self-immunity protein *cvi* were found to be similarly, organized and annotated in two reference strains *Xfp* DD and *Xfm* ESVL (Figure 1).

The three *cvaC* genes are annotated as “colicin V synthesis protein” in both genomes (*Xfm* ESVL: Acc. Nr. CP099516.1; protein_ID: WLE27528.1, WLE27529.1, WLE27530.1; *Xfp* DD: Acc. Nr. CP020870.1; protein_ID: WP_046417857.1, WP_046417859.1, WP_046417863.1) (Giampetruzzi et al., 2017a,2019b; Román-Écija et al., 2023). The *cvi* gene is functionally annotated as a “hypothetical protein” in *Xfp* DD (WP_046417855.1) and it is not annotated in *Xfm* ESVL, although an open reading frame (ORF), underlined with a dashed yellow arrow, is present (Figure 1).

RNA extraction and DNase treatment were performed following the TRIzol™ Reagent protocol (Invitrogen™) and DNase I treatment (Thermo Scientific), respectively; cDNA synthesis was carried out using the M-MLV Reverse Transcriptase protocol (Invitrogen™). RNA fractions were recovered from 7 days old bacterial colonies grown on PD3 medium. The reaction was set up using a 2x Fast SYBR™ Green Master Mix (Applied Biosystems™) according to manufacturer’s recommendations. The following thermal cycling profile was used: 50°C for 2 min, 95°C for 2 min, followed by 40 cycles of 95°C for 15 s, 58°C for 30 s and 72°C for 30 s. Primer sets specificities were confirmed by checking the absence of non-specific amplifications in melting curve analysis, consisting of a continuous 0.5°C increase in temperature from 65 to 95°C. Experiments were repeated in two independent times and each sample was amplified in three technical replicates. The efficiencies of the newly designed primers and the one of housekeeping gene were preliminarily assessed using 10-fold serial dilution of purified bacterial DNA with a known concentration. The stable transcript of the *dnaQ* gene, encoding a DNA polymerase III subunit epsilon, was used as an internal control to normalize the expression of the other genes (Zaini et al., 2008; Wei et al., 2021). The change of the expression levels of the three target genes (*cvaC-1*, *cvaC-2* and *cvi*), compared to the *dnaQ* gene control, was calculated using the 2^–Δ*Ct*^ method (Livak and Schmittgen, 2001). To verify the potential presence of residual DNA after digestion, qPCR reactions without reverse transcriptase enzyme were also set up using the *dnaQ* specific primers.

#### 2.6.2 TaqMan-based RT-qPCR assay for the detection of *cvaC-1* transcript in host plants

Based on multiple alignments of representative *Xfp* and *Xfm* genomes deposited in GenBank (Supplementary Figure 2), performed with Geneious version 2023.2.1, of the first copy (*cvaC-1*) of *cvaC* operon, a primer pair (col_F/R) and a 6′-carboxyfluorescein/Black Hole Quencher-1-labeled probe (6′FAM/BHQ) TaqMan (col_Prb) were specifically designed using NCBI Primer-Blast tool v. 4.1.0 and PrimerQuest™ Tool (Integrate DNA Technology) for a TaqMan based RT-qPCR. Protocols used for plant RNA extraction and RT-qPCR conditions are described in the paragraph below.

#### 2.6.3 Analytical sensitivity and primer efficiency

To assess the diagnostic sensitivity of the new *cvaC-1* RT-qPCR assay and establish its limit of detection (LoD), total RNA recovered from xylem tissues of a *Xfp* infected olive, either with the traditional CTAB method (Loconsole et al., 2014) followed by DNase I-treatment or by using the Maxwell^®^ RSC Plant RNA Kit (as described in paragraph 2.7.1) was used. Primer efficiency and analytical sensitivity were determined by calculating the slope value of the standard curves generated with five ten-fold (from 1 to 10^–5^) serial dilutions of the two digested RNA samples. Each dilution was analyzed in duplicate and the average Cq was calculated. The experiment was repeated in two independent runs. The primer efficiency was computed using the following formula: E = 100 × (10^–1/*slope*^ – 1). The associated Pearson’s product-moment correlation coefficients (PMCC) were also reported. To further support RT-qPCR results, a primer pair for end-point RT-PCR, covering the entire length of the gene, was manually designed for the amplification of the whole *cvaC-1* gene. The diagnostic sensitivity of this assay was assessed by using the same serial dilutions used for RT-qPCR. All primer and probe sequences used in this study are listed in Table 1.

**TABLE 1:** RT-qPCR and RT-PCR primer/probe sequences used in this study.

Primer name	Sequences (5′–3′)	Size (mer)	Molecular analysis
col_F	ACGGGTTCCACCCCAGAT	18	RT-qPCR (TaqMan-based; Gene expression)
col_R	CGGCTTAACGCAGCTATCGT	20	
col_Prb	FAM-CCAGCAAAAAAAGCAGCAACGCCA-BHQ	25	RT-qPCR (TaqMan)
cvaC-2_F	GCAACGTGTCAGGTGGTGATTTTGC	25	RT-qPCR (Gene expression)
cvaC-2_R	CCAGATGGAGCCAGCAAAAAAACC	24	
cvi_F	TCGAGCAGAATCGAAAGGGT	20	
cvi_R	GTTCCCATAGGGACCACTG	19	
dnaQ_F	TGATACTGAGACCACTGGCC	20	
dnaQ_R	CCGGACTCAAACGACACATC	20	
cvaC-1_F	ATGCGTGAATTAACATTGAC	20	End-point RT-PCR
cvaC-1_R	TTACTTAGCGAAGGTGCCGT	20	

### 2.7 Monitoring *in planta* accumulation of *cvaC-1* during the infection process

**FIGURE 2:**
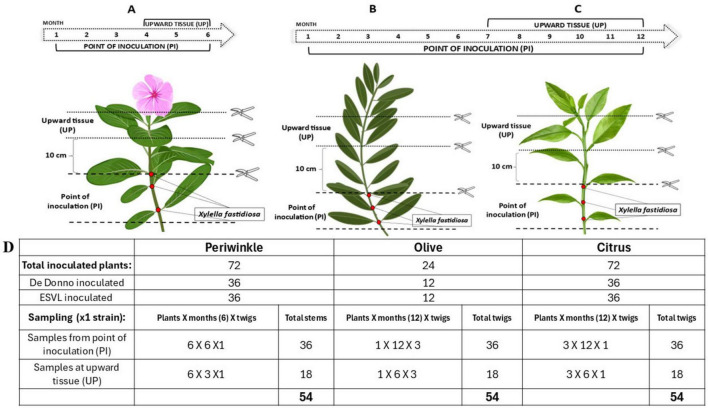
Schematic cartoon representing the time course of *Xf* inoculation experiment. For all the three species (periwinkle, olive and citrus), two consecutive needle-inoculation points (red dots) were made at the basal part of the stem. Six and three stem/twigs, harboring one or two inoculation points (PIs) were collected once per month for the first three and 6 months, respectively from the periwinkle **(A)** and from the olive **(B)** and citrus **(C)** plants. The remaining inoculated stems/twigs were monthly collected, by sampling the PIs and the 10 cm portions above the last inoculation point (UP) during the last 3 and 6 months of the trial for periwinkle and olive and citrus plants, respectively. Overall, 54 tissue samples *per* species *per Xf*-strain were analyzed. **(D)** Summary table of sampled tissue for each species throughout the whole trial.

Two bacterial strains, *Xfp* DD and *Xfm* ESLV were used to inoculate three different plant species and subsequently monitor the bacterial host colonization by standard qPCR assay (Harper et al., 2010) targeting bacterial DNA (Amoia et al., 2023) in comparison with the newly developed TaqMan-based RT-qPCR assay targeting the *cvaC-1* gene. More specifically, the test included 24 olive trees cultivar Ogliarola salentina, 72 periwinkle plants (*Catharanthus roseus* (L.) G.Don, 1837) cultivar Madagascar and 72 citrus plants (Citrus × sinensis (L.) Osbeck, 1,765) cultivar Madam Vinous. Olive is known to be highly susceptible to *Xfp* DD infections while, in previous greenhouse tests, the strain ESLV needle-inoculated in olives proved to be able to colonize the plants to a lower extent than *Xfp* DD and without inducing typical shoot dieback and desiccations (Saponari, personal communication). Two-years-old olive plants with several ramifications were used, which allowed for the inoculation of multiple twigs, thus requiring a lower number of plants compared to the other species. These latter consisted of seedlings grown as single stems. Inoculated plants were grown in a quarantine greenhouse at 25°C ± 2 and relative humidity (RH) of 65% ± 10. For needle inoculation, bacterial suspensions were prepared by dispersing scraped colonies in sterile distilled water and their titers were adjusted to an optical density (OD_600_) of 1.0. Two small drops (8–10 μL each) of the suspension were placed at the level of two consecutive leaf nodes in the basal part of the main stem for citrus and periwinkle and multiple twigs for olive. Drops were pricked 4–5 times with a sterile entomological needle. Overall, experiments on olive and citrus lasted 12 months, while on periwinkle 6 months. Sampling and testing were performed on a monthly basis, starting 1 month after the inoculation, as follows: for the first three (in periwinkle) and 6 months (in olive and citrus) a total of six stem portions, each comprising an inoculation point (samples identified with the suffix PI), were collected and tested. For the remaining experimental period, in addition to the six inoculation points, the portions of the stem located 10 cm above the last inoculation point (samples identified with the suffix UP) were also harvested and tested (Figures 2A, B, C). Conclusively, 54 samples for each strain/species combination were tested (Figure 2D).

#### 2.7.1 Nucleic acids extraction and PCRs conditions

Small pieces of stems/twigs were used to extract total DNAs with the commercial PureFood GMO Authentication Kit and total RNAs with the Maxwell^®^ RSC Plant RNA Kit on an automated extraction platform. Total DNAs were employed to set up qPCR reactions for the amplification of the bacterial *rimM* gene (Harper et al., 2010), using the conditions described by (Amoia et al., 2023). Whereas total RNAs, previously digested with DNAse I/RNAse free, were tested by RT-qPCR and RT-PCR using the primers designed in this work (Table 1).

Briefly, 500 mg of tissue was homogenized in the presence of commercial CTAB (Cetyltrimethylammonium bromide) buffer added with chilled 2% 1-thioglycerol and heated at 65°C for 15 min; the clear homogenate was loaded in the cartridge according to manufacturer’s instructions, including the step of the DNase I treatment. The eluted RNAs (50 μL) were stored at −80°C until processing. For *cvaC-1* quantitative amplification, the optimal primer/probe concentrations in the reaction mix were determined as those yielding the highest reporter fluorescence (RFUs) and the lowest threshold cycle (Ct). To establish the best annealing temperature of the new primer/probe set, a temperature gradient was carried out on a range spanning from 58 to 63°C. One-step RT-qPCR reactions were performed in a final volume of 11 μL containing 5.5 μL 2x iTaq Universal Probes Reaction Mix (Bio-Rad, Hercules, CA, USA), 0.3 μL each forward and reverse primer (10 μM; col_F/R), 0.2 μL FAM-labeled probe (col_Prb), 0.2 μL iScript Reverse Transcriptase (RNAse H ++ MMLV enzyme), 1 μL (up to circa 100 ng μL^–1^) of digested RNA sample and nuclease-free water to the final volume. Each sample was amplified in duplicate and the average Cq was considered. All qPCR and RT-qPCR analyses were run in a Bio-Rad CFX96 Real-Time PCR Detection System (Bio-Rad, Hercules, CA, USA). Data acquisition and analysis were performed using the CFX Maestro 1.1 version N. 4.1 software (Bio-Rad, Hercules). The threshold line was automatically set by the software or manually adjusted when necessary. To carry out RT-PCR, cDNA was first synthesized using the above-mentioned standard reverse transcription protocol. Then, PCR reaction mix was set up as follows: 2x GoTaq^®^ Green Master Mix, 0.5 μL of each primer (cvaC-1_F/R), 1 μL of cDNA and nuclease-free H_2_O to 20 μL final volume. The PCR program included: one cycle at 95°C for 3 min, 35 cycles at 94°C for 30 s, 58°C for 30 s, and 72°C for 30 s, followed by a final extension at 72°C for 5 min.

#### 2.7.2 Re-isolation in pure culture of bacterial strains

A few of the periwinkle plants displaying systemic infections, upon the inoculation of both strains (*Xfp* DD and *Xfm* ESLV), were used for re-isolation of the inoculated bacterial strains 6 months after the inoculations, before ending the experiment. Systemic infection was assessed by testing tissues distant from the inoculation points that gave positive results in qPCR for the detection of *Xf* (Montilon et al., 2022). Stem pieces were washed under tap water, surface-sterilized in 2% sodium hypochlorite for 3 min, soaked in 70% ethanol for 3 min and rinsed three times in sterile water. Cutted smaller pieces were automatically macerated in an extraction bag (Bioreba) in presence of sterile 1x Phosphate Buffered Saline (PBS) buffer (0.05 M NaCl, pH 7.2) and three consecutive ten-fold dilutions of the recovered sap were plated on periwinkle wilt gelrite (PWG) agar plates (Hill and Purcell, 1995). Plates were incubated for 3–4 weeks at 28°C and periodically inspected for the growth of *Xf* colonies.

### 2.8 Validation of the *cvaC*-based detection assays in chronically infected and symptomatic olive plants

Systemically infected olives (i.e., olives where *Xf* was detected by qPCR also in tissues far from the points of inoculations) were used to test the reliability of the RT-PCR and RT-qPCR detection assays for the identification of bacterial RNA and discriminate between tissues supporting active bacterial multiplication (i.e., infected vegetating shoots) and those harboring non-growing cells, likewise infected desiccated plant tissues in which the bacterial cells are not sustained any multiplication process, as *Xf* is not a saprophytic bacterium and requires living plants, specifically the xylematic system, to survive and cause disease. Infected plants were obtained by grafting onto healthy 2 years old olive plants (cv Ogliarola salentina), cuttings collected from field naturally infected trees of the same cultivar. Upon graft taking and sprouting, the occurrence of the bacterium in the new shoots was assessed, and 48 plants yielding qPCR-positive reactions were retained for the experiment, along with 6 *Xf*-free plants of the same cultivar. Six of the 48 plants were treated with tetracycline by trunk injection, delivering the antibiotic directly to the xylem where *Xf* multiplies, to assess the impact of the antibiotic on the bacterial multiplication rate as well as the efficiency of the newly developed *cvaC-1*-based assays to monitor the effect of such applications. Previous *in vitro* and *in planta* studies have shown that tetracycline could inhibit *Xf* growth leading to a reduction or temporal suppression of symptoms in treated plants, but none of the treatments eliminated the bacterium permanently (EFSA Panel on Plant Health (PLH), 2016). Briefly, at 12 months after grafting, corresponding to 6 months after the application of tetracycline for the treated plants, when approximately half of the plants started to show mild to severe shoot dieback and desiccation (Supplementary Figure 3), samples consisting of small pieces of twigs were collected. In the case of symptomatic plants, these consisted of desiccated, withered, and dried tissues. The DNA and RNA fractions were recovered as previously described. An aliquot of the DNA was used to set up the qPCR assay for the conventional detection of the bacterium, whereas an aliquot of the purified and DNA-digested RNA was used to arrange the two-step RT-PCR and the one-step RT-qPCR reactions, both targeting the *cvaC-1* transcript.

### 2.9 Data analysis

Regression analysis for analytical sensitivity was performed in R environment, using Rstudio version 4.3.1. According to the normal distribution of results, parametric (Paired *t*-test) or non-parametric (Wilcoxon Signed Rank Test) analysis was performed between qPCR and RT-qPCR Cq data in SigmaPlot v.15.0. *P*-values connected to the significance of both tests are also reported. GraphPAD Prism 9.3.0 software was also employed to perform the correlation analysis and the Principal Component Analysis (PCA) of qPCR/RT-qPCR results for tetracycline-treated/non-treated samples, and generate whisker plots with Cq data obtained from qPCR/RT-qPCR on artificially inoculated plants. The correlation between RT-qPCR *cvaC-1* and qPCR by Harper et al., 2010 was determined using Spearman’s correlation coefficient.

## 3 Results

### 3.1 Colicin V precursor is highly transcribed in infected olive

**FIGURE 3:**
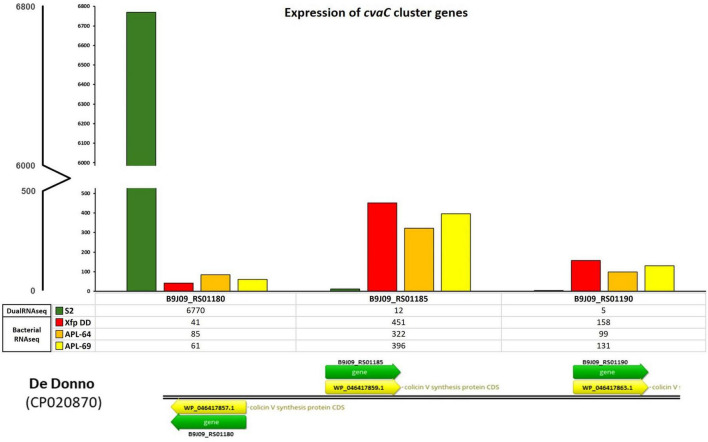
*cvaC*-operon transcripts from bacterial RNASeq and dual RNA-seq. Expression of *cvaC* genes (*cvaC-1*, *cvaC-2*, *cvaC-3*) *in planta* from dual RNA-seq (S2) and *in vitro* from the RNASeq of *Xf* cultured strains (Xfp DD, APL-64 and APL-69). The numbers of normalized reads of the three genes in the operon and the legenda are reported on the y-axis

The olive tree S2, displaying the lowest quantification cycle (Cq) value (< 20, corresponding to a bacterial population in the range of 10^7^–10^6^ CFU/mL) among the 12 screened by qPCR, was selected for the total RNAs extraction and the construction of the dRNA-seq library. The sequenced library generated 40,261,113 raw reads. After the quality check, 31,407,417 (78%) reads were aligned to the olive genome (cv. Farga v9) and the resulted unmapped reads (8,853,696) were aligned to the *Xfp* De Donno (*Xfp* DD) reference genome (CP020870), resulting in 21,411 aligned reads to the bacterial genome (sequencing reads available from NCBI BioProject ID PRJNA1141272) (Table 2). Reads mapped to the *Xfp* DD genome were used to determine the abundance of annotated bacterial transcripts which consisted of 2,319 genes (GCF_002117875.1_ASM211787v1_genomic.gff.gz) including 6 ribosomal RNAs (rRNA), 49 transfer RNAs (tRNAs), 3 non-coding RNAs (ncRNAs), 1 transfer-messenger RNA (tmRNA) and 2,260 coding protein sequence (CDS). The raw counts were normalized to 20,000 mapped reads to obtain expression values comparable to the bacterial RNASeq data set obtained from the *in vitro* grown *Xf* cultures. Only 125 out of the 2,319 annotated genes were covered by a number of mapped reads greater than 10, and only 10 genes were covered by over 100 reads (Supplementary Table 1, mapped reads in dual RNA-seq; S2 column). Unexpectedly, the gene with the highest number of mapped reads (6,770 normalized counts) codes for a “colicin V precursor” (B9J09_RS01180, i.e., the first gene in the *cvaC* operon of *Xf*, indicated in the text as *cvaC-1)*, and was much more expressed than the *rnpB* gene (B9J09_RS09380), an essential bacterial catalytic RNA (RNase P) known to be highly transcribed (Parker et al., 2016; Ellis, 2020) (Figure 3 and Supplementary Table 1).

**TABLE 2:** Statistics on RNASeq sequencing libraries (DualRNA-seq and bacterial RNASeq).

Library	–	Raw reads	Mapped reads on olive genome (%)	Unmapped reads to olive genome	Mapped reads on *Xf* De Donno genome
Dual RNA-seq	S2	40,261,113	31,407,417 (78%)	8,853,696	21,411
Bacterial RNASeq	Xfp DD	44,798,814	–	–	31,721,799
	APL-64	40,463,402	–	–	28,505,044
	APL-69	46,980,188	–	–	33,586,808

### 3.2 RNA sequencing on *in vitro* cultured strains of *Xylella fastidiosa*

To extend the dataset of bacterial gene expression profiles, three RNASeq libraries were constructed and sequenced starting from total RNAs extracted from three cultured strains of *Xfp* ST53 (Xfp DD, APL-64, APL-69; NCBI BioProject ID PRJNA505446) grown *in vitro* on PD3 agar plates. High-throughput sequencing of these three libraries generated 44,798,814, 40,463,402 and 46,980,188 raw reads, respectively for Xfp DD, APL-64, APL-69. After the quality check, 31,721,799 (Xfp DD), 28,505,044 (APL-64) and 33,586,808 (APL-69) reads were mapped to the *Xfp* DD reference genome (CP020870) with an average coverage of 1000X (sequencing mapped reads available from NCBI BioProject ID PRJNA1141272) (Supplementary Table 1, mapped reads in Bacterial RNASeq columns). Beside the *rnpB* (B9J09_RS09380) and *ssrA* (small stable RNA A; B9J09_RS11595) genes, the gene B9J09_RS06930 annotated as Blp family class II bacteriocin, was the highest expressed transcript with 505, 476 and 455 normalized reads in the three RNASeq libraries (Supplementary Table 1, mapped reads in Bacterial RNASeq columns). The number of reads mapping *cvaC-1* “colicin V precursor gene” (B9J09_RS01180), found to be overexpressed *in planta*, was approximately ten times lower, ranging from 41 to 85. By contrast, the number of reads mapping the *cvaC-2* gene, the second gene of the *cvaC* operon, was much higher and similar to that of the most expressed *in vitro* genes (between 322 and 451 reads) (Supplementary Table 1, mapped reads in Bacterial RNASeq columns, Figure 3).

### 3.3 Validation by RT-qPCR of the expression of *cvaC* genes in cultured bacterial strains

**FIGURE 4:**
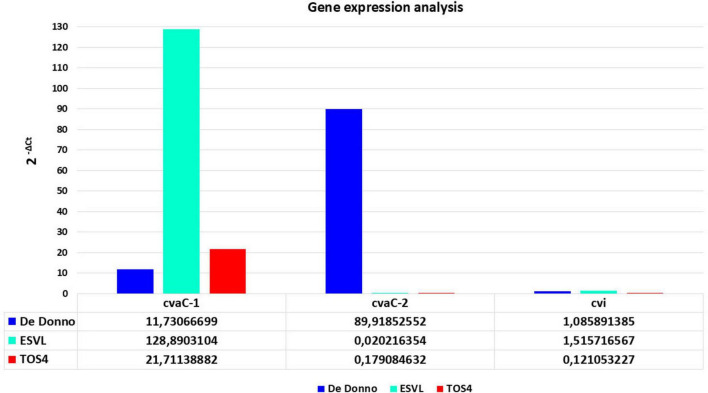
RT-qPCR gene expression analysisof *cvaC-1*, *cvaC-2* and *cvi*,of *in vitro* grown bacterial strains of *Xf* (*Xfp* DD, *Xfm* ESVL and TOS4). The 2^– ΔCt^ expression values of the three selected genes, compared to the stable one of housekeeping gene *dnaQ*, are shown on y-axis. RT-qPCR reactions were done in triplicate.

The RT-qPCR results showed that *cvaC-1* transcript is upregulated under the *in vitro* culturing conditions for the three strains, although its expression level (fold changes) was low in *Xfp* DD and *Xfm* strain TOS4 and higher in *Xfm* strain ESVL (Figure 4). Conversely, *cvaC-2* transcript was over-expressed in *Xfp* DD compared to both *Xfm* cultured strains. The RT-qPCR results for these target genes confirmed the RNASeq data on the *Xfp* strains, with the expression (2^–Δ*Ct*^) ratio between *cvaC-2* and *cvaC-1* (7,6653) almost overlapping the average ratio of the reads *cvaC-2*: *cvaC-1* recovered from RNASeq (7,0722) for the three *Xfp*-cultured strains Xfp DD, APL-64 and APL-69. For the strains tested, a low expression level was consistently detected by RT-qPCR for *cvi* gene (Figure 4). Direct qPCR amplification of the RNA extracts produced high quantification cycle (Cq) values (> 35), indicating that the RNA templates did not contain any residual DNA traces.

### 3.4 *In planta* confirmation of the expression of *cvaC-1* transcript

**FIGURE 5:**
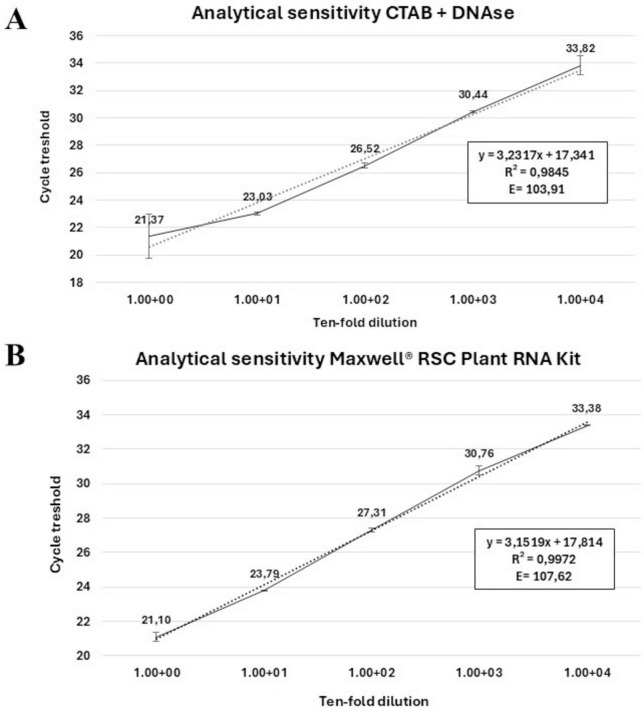
Analytical sensitivity of TaqMan-based RT-qPCR. Linear regression curves of the RT-qPCR assay targeting the *cvaC-1* transcript. Standard curves were generated using 10-fold dilution series of the total RNA extracted from a *Xfp* infected olive twig purified with **(A)** a CTAB-based protocol with DNA digestion and **(B)** the RSC Plant RNA kit. Quantification cycles on the y-axis are correlated to the log of dilutions on the x-axis. In both figures, the equation regression, Pearson’s correlation values (*R*^2^) and % primer efficiency are also reported.

The primer pair col_F/R, amplifying a 69 bp fragment, coupled with a TaqMan probe (col_Prb) successfully detected the *cvaC-1* transcript by RT-qPCR assays in artificially inoculated plants at different time points post-inoculation. The optimal annealing temperature was defined at 59°C. Thus, the selected RT-qPCR parameters were as follows: an initial reverse transcription step at 50°C for 10 min; polymerase activation and DNA denaturation at 95°C for 3 min followed by 40 cycles of denaturation at 94°C for 15 s and annealing at 59°C for 30 s. No reaction amplifications were observed in negative internal control (NIC) and no template control (NTC). When Cq values were plotted against the logarithm of the dilutions, standard curves with very good linearity and high confidence level, having *R*^2^ values of 0.9845 and 0.9972, respectively using the RNA recovered by the CTAB-based method and with the Maxwell^®^ kit, were generated (Figures 5A, B). In both cases, the efficiencies recorded (103.91 and 107.62%) fit in the optimal range of 90–110% (Bustin et al., 2009). These values are similar to those previously reported for the qPCR assay (Amoia et al., 2023). High Pearson’s correlation values (0.9922 and 0.9986, for RNA CTAB-based and Maxwell^®^ RSC Plant RNA kit extracted, respectively) were achieved. The linear regression equations associated with calibration curves are displayed in Figures 5A, B. In addition, primers cvaC-1_F/R designed to amplify the whole gene in RT-PCR, yielding a 309-bp amplicon, successfully identified the entire transcript in systemically infected plant tissues, although as expected, the diagnostic sensitivity of the assay was lower than the RT-qPCR test (Supplementary Figure 4). Even so, this assay proved to be highly specific in identifying *cvaC-1* intact and complete transcripts, such as those associated with bacterial multiplication under optimal conditions. Indeed, negative results (no amplifications) were consistently obtained with RT-PCR (i.e., amplifying the whole gene) even if the same total RNAs purified from desiccated tissues, where the bacterial growth and multiplication could be suffering, generated very high Cq values (> 30) in RT-qPCR.

### 3.5 *cvaC-1* detection in artificially inoculated plants

**FIGURE 6:**
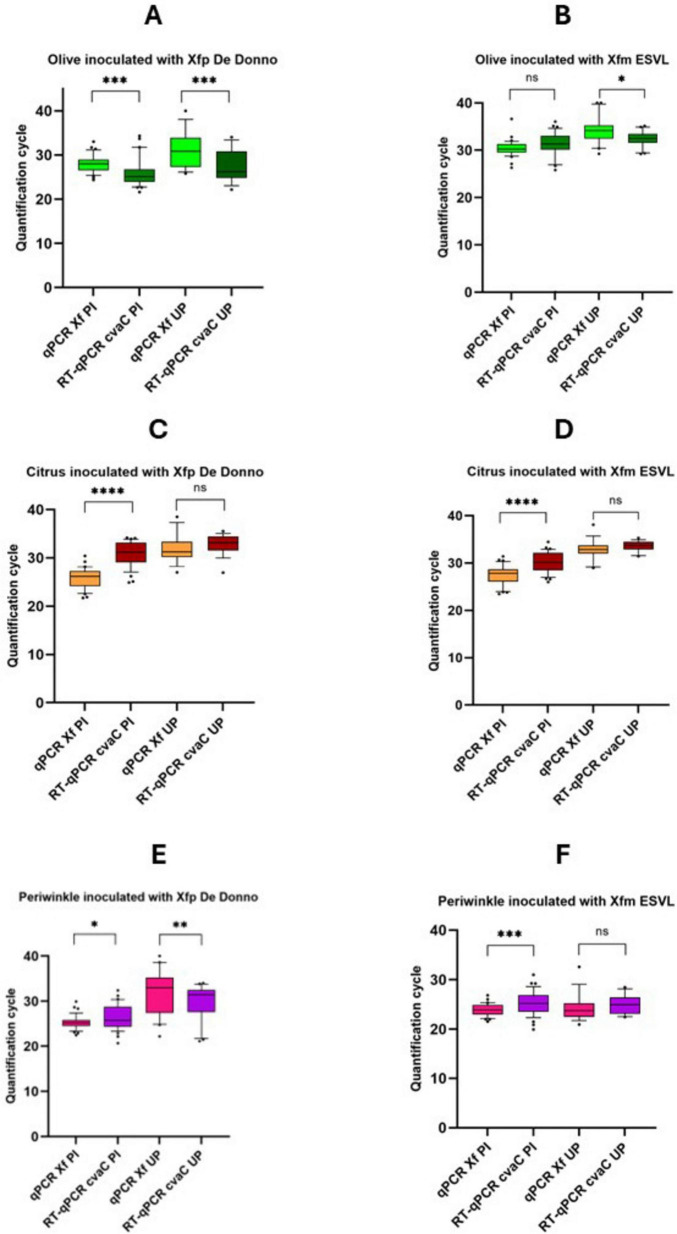
Whisker plots of qPCR and RT-qPCR results. Data are recovered from olive **(A,B)**, citrus **(C,D)**, and periwinkle **(E,F)** plants artificially inoculated with Xfp DD and Xfm ESVL bacterial suspension (OD600 = 1). Molecular analyses were performed using purified nucleic acid extracts recovered both from stem tissue comprised by two consecutive inoculation points (PI) and twig/stem portions 10 cm far from the last inoculation point (UP). Quantification cycles are indicated on the *y*-axis. The statistical significance level is also reported above each thesis (****p < 0.0001; ***p < 0.001; **p < 0.01; *p < 0.05; ns, not significant).

Altogether, 54 samples/strain (in total 108 for each species) were analyzed during the 6 and 12 months of the trials, respectively for periwinkle, and olive and citrus. Results of the qPCR and RT-qPCR assays for each plant species (olive, citrus and periwinkle) and samples (PI and UP) are reported in detail in Supplementary Figure 5, while Cq values, cumulative from all sampling times, are graphically reported in Figure 6. In *Xfp* DD-infected olives (Figure 6A), the average Cq values obtained by RT-qPCR were always lower than those recovered by qPCR assay, both for the samples collected at the point of inoculation (samples identified with the suffix PI) (25.69 ± 3.54 *vs* 28.01 ± 1.88; *P*-value < 0.001) and 10 cm above the last inoculation point (samples identified with the suffix UP) (27.74 ± 4.64 *vs* 31.36 ± 3.92; *P*-value = 0.00161). The opposite was observed in *Xfm* ESVL-infected olives where the average Cq values of infected sample “PI” were 31.38 ± 2.98 *vs* 30.38 ± 1.72, with no significant difference between them, and those from “UP” samples corresponding to 32.27 ± 1.74 *vs* 34.30 ± 2.88 (*P*-value = 0.008), respectively in the RT-qPCR *cvaC-1* amplification *vs* qPCR (Figure 6). These findings denote that *Xfp* DD replicates at higher rate than *Xfm* ESVL in olive trees and corroborate the results of previous works (Nunney et al., 2019); Saponari, personal communication) indicating a reduced rate of olive colonization and pathogenicity of *Xfm* ESVL in olives. Indeed, Nunney et al. (2019) reported that enzyme-linked immunosorbent assay (ELISA) does not detect any positive olive inoculated with *Xfm* strain “Dixon,” which belongs to the same ST (ST6) of ESVL used in this study. Citrus species have been proved to be immune to *Xfp* ST53-isolate and no report of infections caused by strains of the subspecies *multiplex* have been so far recorded. The results of our experiments are consistent with the predicted immunity to both bacterial strains. More specifically, samples collected at the PI yielded low Cq values in conventional qPCR, i.e., 25.76 ± 2.10 and 27.51 ± 2.00 for *Xfp* DD and *Xfm* ESVL inoculated plants, respectively, while generated high Cq values by RT-qPCR targeting *cvaC-1* (*Xfp* DD 30.89 ± 2.60 *vs Xfm* ESVL 30.20 ± 2.21), indicating that no active bacterial multiplication occurred at the inoculation points following the needle inoculations and that the low Cq values generated in qPCR corresponded to the detection of the cells of the inoculated bacterial suspensions. When the portions collected 10 cm above the point of inoculations were tested, Cq values close to the detection limit were obtained both by qPCR (31.95 ± 2.91 for *Xfp* DD and 32.86 ± 2.21 for *Xfm* ESVL) and RT-qPCR (32.87 ± 2.07 for *Xfp* DD and 33.60 ± 1.12 for *Xfm* ESVL). Again, the Cq values recorded in RT-qPCR for *cvaC-1* were higher than those obtained for the conventional qPCR assay, suggesting the lack of an active multiplication of the bacteria. Moreover, the observed high Cq could be ascribed to the passive translocation of the inoculated bacterium through the xylem flux. Periwinkle confirmed its high susceptibility to the bacterium. For both strains, Cq values obtained at the inoculation points in qPCR were close or lower than 26; while the Cq values generated in RT-qPCR were close or lower than 28, with a *P*-value = 0.029 *vs* < 0.001. Tests on the UP portions of the plants showed different results for the two bacterial strains. Plants inoculated with *Xfm* ESVL strain generated low and equivalent Cq values in qPCR (24.36 ± 2.73) and RT-qPCR (24.94 ± 1.91). Conversely, the comparison of qPCR and RT-qPCR results (32.03 ± 5.01 *vs* 29.89 ± 3.84; *P*-value = 0.010) showed that periwinkle is a less conducive host for *Xfp* DD (Figure 5). Indeed, re-isolation of both bacterial strains was successfully achieved from the distal part of the inoculated plants (UP), obtaining 1.05 × 10^5^ and 5.9 × 10^3^ CFU/mL, respectively from *Xfm* ESVL and *Xfp* DD infected periwinkles.

### 3.6 Validation of the *cvaC*-based detection assays to differentiate growing and non-growing bacterial cells

Regardless of the vitality of the plant tissues (desiccated or vegetating twigs), positive qPCR reactions, targeting the bacterial DNA, were generated for all 42 infected olive plants. More than 50% of the non-tetracycline-treated plants (19 out of 36) had developed shoot dieback and desiccation (Supplementary Figure 3, Supplementary Table 2). As expected, in the samples collected from these plants, Cq values were higher, but still indicating a relevant bacterial DNA accumulation for the withered/desiccated tissues than for the green asymptomatic cuttings (25.18 ± 1.38 *vs* 20.70 ± 0.91). Values above the Cq negativity threshold were observed in the six *Xf*-free control plants.

In contrast, the Cq values generated by the *cvaC-1* RT-qPCR were significantly different, with an average of 16.65 ± 1.14 for the symptomless plants (i.e., viable and green twigs) and an average Cq of 29.80 ± 1.43 for the desiccated twigs (Supplementary Tables 2, 3). Remarkable results were recorded on the six tetracycline-treated plants which showed broader variation compared to the untreated ones for conventional qPCR and RT-qPCR, respectively (Supplementary Table 3), suggesting that the treatment impacted cell vitality in some plants. Indeed, this issue is also supported by the associated coefficients of variation, which were 13.04% for standard qPCR and 25.35% for RT-qPCR. Only one of these plants showed shoot dieback, and consistently with the results on the non-treated plants, high Cq values (qPCR: 30.56; RT-qPCR: 31.87) were obtained in both assays on the sampled tissues. The remaining five treated and asymptomatic plants could be differentiated into two groups. The first included three plants in which the results were in line with those recorded in vegetating non-treated plants (qPCR: 22.52 ± 0.95; RT-qPCR: 19.05 ± 0.72), thus supporting high bacterial populations and intact *cvaC-1* transcript, as demonstrated by the low Cq values in both assays and the positive RT-PCR results with a clear band in agarose electrophoresis gel. The second group encompassed the remaining two asymptomatic antibiotic-treated plants, whose diagnostic results were more similar to those recorded for the desiccated tissues harboring non-growing bacterial cells (qPCR: 25.94 ± 0.31; RT-qPCR: 29.05 ± 2.73), including the lack of amplification in RT-PCR, indicating that the bacterial growth was affected by the antibiotic treatment and its effect could be measured with the tests herein developed.

These results confirm that *cvaC-1* transcript accumulates in host tissues with active bacterial multiplication, while desiccated tissues, in which the bacterial multiplication is compromised, generate high Cq values, most likely related to low levels of residual and degraded RNAs. By comparing the qPCR and RT-qPCR results, it appears that when the bacterium is actively colonizing the plant tissues, the Cq values of the RT-qPCR are consistently lower than those obtained using the conventional qPCR targeting the bacterial DNA (*rimM* gene). Whereas under conditions not favorable for bacterial multiplication and survival, such as in withered and desiccated tissues, RT-qPCR generated Cq values higher than those recorded in qPCR.

**FIGURE 7:**
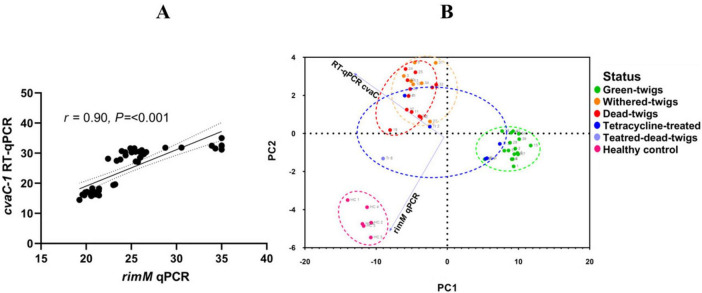
Principal component analyses (PCA) of *Xfp* infected olives. **(A)** Scatter plot depicting the Spearman correlation between the RT-qPCR *cvaC-1* and conventional qPCR assays. The strong positive correlation indicates that both methods consistently detect and quantify bacterial genetic material across different sample types. **(B)** PCA biplot representing the clustering of olive samples based on Cq values from RT-qPCR *cvaC-1* and qPCR. Samples are color-coded according to their condition: green-twigs (green), withered-twigs (orange), dead-twigs (red), treated (blue), treated-dead-twigs (light blue), and healthy control (pink). The first principal component (PCA1) accounts for 91.75% of the total variance, highlighting distinct group separation based on bacterial viability and treatment effects.

Clear-cut results were obtained through RT-PCR. A single DNA band of the expected size was observed in all vegetating shoots tested (Supplementary Table 2), while samples consisting of desiccated and dead tissues did not show any band in the electrophoresed agarose gel. More precisely, no DNA bands were detected in samples that generated Cq values higher than 25 in RT-qPCR assays. Such discrepancy can be attributed most likely to the occurrence of degraded and fragmentary RNA in these tissues, whose small fragments were still detected by RT-qPCR, while the amplification of the whole transcript was impaired, rather than to the lower analytical sensitivity of the RT-PCR compared to the RT-qPCR (Supplementary Figure 4). Further statistical evaluation of the relationships between conventional qPCR and *cvaC-1* RT-qPCR results occurring among the analyzed olives are shown by Spearman’s correlation and PCA (Figures 7A, B). The correlation graph (Figure 7A) shows a strong relationship (*r* = 0.90; *P* < 0.001) between the Cq values of RT-qPCR for the *cvaC-1* gene and those of the conventional qPCR. The PCA clearly clusters Cq data from green-twigs (vegetating infected twigs), withered-twigs (withered infected twigs), dead-twigs (dead infected twigs), treated (twigs from infected plants treated with tetracycline HCl), and healthy control (twigs from non-infected plants). The results show that the first principal component (PC1) has an eigenvalue of 62.49, explaining about 91.75% of the total dataset variance. The second principal component (PC2) has an eigenvalue of 5.62, explaining 8.25% of the variance, meaning that a single direction (PC1) captures almost all the information present in the data, indicating an excellent simplification of the variables present in the data set (Figure 7B).

Overall, the estimation of the accumulation of the *cvaC-1* transcript in host plants throughout the infection process (Supplementary Figure 5) and in chronically and symptomatic tissues, consistently showed that: (i) with highly susceptible host-bacterial strain combinations (i.e., olive - *Xfp* DD; periwinkle - *Xfm* ESLV) or moderately susceptible combinations (i.e., olive - *Xfm* ESLV; periwinkle - *Xfp* DD) detection of *cvaC-1* transcripts consistently displayed equal or, more frequently, lower Cq values than those generated by qPCR targeting the bacterial DNA; (ii) when incompatible host-bacterial strain combinations (i.e., citrus - *Xfp* DD; citrus - *Xfm* ESLV) were examined, the average Cq values recorded for the *cvaC*-*1* transcript where > 30, differently from those observed in the aforementioned combinations, and consistently higher than those generated in the qPCR for the DNA amplification of the *rimM* gene. These results emphasize that this host species was not conducive to the bacterial multiplication, with amplifications being correlated to the initial inoculum source or to the initial colonization of a few primary xylem vessels in which *Xf* was entrapped after inoculation as reported in Niza et al. (2015).

## 4 Discussion

Transcriptomic approaches are widely exploited to investigate either the host or the pathogen gene expression profiles to gather insights into the infection process and the host defense mechanisms. Preliminary RNASeq studies, using total RNAs from *Xfp*-infected olives (Giampetruzzi et al., 2016), did not allow to obtain data from the bacterial gene expression *in planta*, since bacterial mRNAs have short polyA tails which represent a small fraction of the plant total RNAs (Sarkar, 1997), thus not detectable using the conventional RNASeq approach. The dual RNASeq technology, by depleting the large amount of plant and prokaryotic ribosomal RNAs, allows the enrichment of the remaining total RNAs, including the bacterial mRNAs. In the present work, we took advantage of this technology to study the gene expression profile of *Xfp* ST53 colonizing olive trees. In addition, gene expression profiles were also produced for three *in vitro* cultured strains previously recovered from OQDS-affected olives (Sicard et al., 2021). The analysis of the combined transcriptome datasets unraveled novel features of the bacterial lifestyle in real and artificial environments. As expected, the coverage of the bacterial genome was much higher for the transcriptomes from pure bacterial cultures than that obtained by dRNA-seq from total RNAs performed on a selected highly infected olive tree.

The gene expression profile of the bacteria grown *in vitro* showed an elevated expression level of gene B9J09_RS06930, annotated as Blp family class II bacteriocin (GO:0042742 - defense response to bacterium). Class II bacteriocins, most likely the best characterized and the most distributed group in food-associated lactic acid bacteria (Zhang et al., 2022), are synthesized as pre-bacteriocins containing an N-terminal leader sequence that is cleaved during secretion resulting in a small protein (< 60 amino acids) which is heat-stable. A typical gene cluster involved in the biosynthesis of class II bacteriocins consists of genes that encode a bacteriocin precursor peptide, an immunity protein, and an ATP-binding cassette (ABC) transporter. We did not find the associated immunity and ABC genes in the *Xfp* DD genome, where this bacteriocin gene is embedded in a genomic region surrounded by prophage-related genes (data not shown), likely originating from an event of phage-mediated horizontal gene transfer (Touchon et al., 2017).

*In planta*, the dual transcriptome profile unraveled abundant levels of transcript of a colicin-like precursor protein (colicin V), encoded by the *cvaC-1* gene. This protein belongs to the large family of bacteriocins, the most abundant antimicrobial molecules largely conserved across the main bacterial lineages (Riley and Wertz, 2002). Unlike other antimicrobial molecules and antibiotics, they exhibit a narrow spectrum of activity, often limited to members of the same species as the producer, with which it competes in the ecological niche (Riley, 2011). Because of their limited range of activity and moderately few side effects, bacteriocins could represent an attractive option for plant disease control and inspire the design of antimicrobial molecules for medical and agricultural use. Notably, those found in *Xf* genomes are mainly PFT and constitute a putative virulence factor of the bacterium, thus contributing to pathogen invasion, systemic spread and biofilm formation (Pashalidis et al., 2005; Zaini et al., 2008) in xylem vessels. From the structural point of view, the C-terminal domain harbors both the cytotoxic activity and the specific site for the immunity protein *cvi*, whereas the presence of a double-glycine-type leader sequence, often associated with some bacterial peptide antibiotics, most likely could be engaged in the quorum sensing mechanism. Our work unveiled that *cvaC-1* and *cvaC-2* microcins have a major role in the *Xf* life cycle, being among the most expressed genes either *in planta* or *in vitro*. Indeed, the differential transcription levels detected between the two investigated conditions indicate that the expression of the two genes is modulated by different environmental conditions like pH and temperature, and factors e.g., nutritional stimuli or the competition with other sensitive bacteria as a strategy to eliminate competitors and improve survival. Moreover, their high expression and toxin production rate *in vitro* may explain the low number of contaminants (i.e., endophytic bacteria) growing on the artificial media when isolating *Xf* in axenic conditions from plant matrices, with *Xf* having a competitive advantage over other bacterial species thriving in the xylem environment. Interestingly, the two *Xfm* strains (ESVL and TOS4) showed a different level of expression of the two *cvaC* genes when grown *in vitro*, underlining different biological features for strains belonging to the same subspecies but harboring different sequence types. A differential transcription level has been also reported in the three *in vitro* grown *Xfp* cultures (Xfp DD, APL-64, APL-69) for the Blp bacteriocin class (Dawid et al., 2007; Fontaine et al., 2007).

The high *cvaC-1* transcription level detected in *Xfp-*infected olives prompted to explore its use as a replication biomarker, i.e., an indicator of the presence of actively growing cells and *Xf* multiplication in plants. To test this hypothesis, we monitored the level of *cvaC-1* transcript in time course artificial infection experiments using strains of two *Xf* subspecies (i.e., *Xfp* DD and *Xfm* ESVL) in three different plant species (olive, citrus and periwinkle). According to previous studies, citrus is renowned for being immune to both strains; on the contrary, periwinkle is susceptible to both, with the Spanish isolate being more aggressive on this host (Delbianco et al., 2021). The olive cultivar Ogliarola salentina is highly susceptible to *Xfp* DD, developing severe canopy desiccations, often leading to the death of the infected trees; while needle-inoculation of the *Xfm* ESVL strain, even if resulted in systemic infections, did not cause shoot dieback and desiccation phenomena, whose transcriptomes profiles did not display overexpression of defense related-genes (Giampetruzzi personal communication). As reported by Nunney et al. (2019), also the *Xfm* strain “Dixon,” which shares the same ST as ESVL used in this study (i.e., ST6), does not induce leaf symptoms and is not able to colonize olive stems, producing negative results in ELISA (Nunney et al., 2019).

Experiments aimed to compare the performances of qPCR targeting the *rimM* region (Harper et al., 2010) and RT-qPCR assay to detect the *cvaC-1* bacterial transcript in the same *Xf*-infected plants, Currently, qPCR represents the golden standard approach for bacterial detection, being the official regulated method in the EU. However, this assay while detecting with high diagnostic sensitivity the bacterial DNA, fails to discriminate between living and dead bacterial cells. Therefore, the developed RT-qPCR assay, targeting the *cvaC-1* transcript, was optimized to overcome this significant limitation.

In host plants where the inoculated strains did not develop systemic infections, low levels of *cvaC-1* transcript were detected, with high Cq consistently above the values recorded in qPCR targeting the *rimM* gene, suggesting that these positive qPCR reactions most likely were related to residual non-growing bacteria cells, i.e., those inoculated but not able to initiate the infection process due to the incompatibility with the host species or the activation of strong defense responses, or unfavorable environmental conditions occurred upon the inoculation events.

Conversely, when host plants harbor active growing colonies, the estimated level of expression of the *cvaC-1* transcript either overlapped the bacterial DNA content or was consistently higher, confirming the overexpression of the target gene during the bacterial host colonization regardless of the strain and the plant species. However, a further feature characterizing this susceptible condition, is the range of Cq values consistently below 30 for both assays, indicating an increased bacterial titer compared to the initial inoculum used to infect the plants.

As expected, in *Xfp* DD-infected olives, both qPCR and RT-qPCR detected a more copious presence of *Xfp* active cells at PI and UP portions compared to the plants infected by *Xfm* ESVL. Conversely, for periwinkle a higher prevalence of growing *Xfm* ESVL cells was detected in all sampled tissues compared to those of *Xfp* DD inoculated plants.

The lack of primary infections in the PI sites of the inoculated citrus plants was evident from the comparison of the Cq values generated by qPCR which were almost ten times (10x) lower than those generated by RT-qPCR, for *Xfp* DD and *Xfm* ESVL strains. As reported in other studies, the lignification process of primary xylem cells is a phenomenon characterizing the resistant genotypes (Coletta-Filho et al., 2007; Niza et al., 2015) with the bacterium remaining confined in the inoculation sites and triggering the secretion of defense compounds that lead to bacterial death (Rodrigues et al., 2013).

Tests performed on the chronically infected plants, showing desiccated olive shoots highlighted two important aspects: (i) conventional qPCR produced strong positive reactions (i.e., Cq < 25) even with dead bacterial cells and processing non-optimal plant tissues, indicating that bacterial DNA persists for prolonged periods, also in dead plant tissues remaining detectable most likely as small fragments of degraded DNA sequences; (ii) although bacterial mRNAs have a short half-life spanning from seconds to hours (Sheridan et al., 1998; Rauhut and Klug, 1999; Laalami et al., 2014), because the RT-qPCR targeted a very short sequence of the *cvaC-1* transcript (69bp), positive reactions occurred even with degraded RNA fractions, as showed by the Cq values recorded for the desiccated shoots tested. Conversely, when these samples were tested by RT-PCR amplifying the whole *cvaC-1* transcript, none of the RNA fractions recovered from dead twigs produced detectable amplification bands. This discrepancy further confirms that RNA is still detectable by RT-qPCR as small fragments even in non-growing cells. However, this was not the case for the whole target transcript, whose integrity was compromised in the desiccated tissues, and as expected, the lack of bacterial multiplication resulted in negative amplification reactions. Although very preliminary and based on a limited number of plants, the efficacy of the application of an antibiotic product on the bacterial multiplication in the plants was clearly detected with our tests, being able to differentiate plants in which a positive effect could be recorded from those in which most likely, the application did not have any impact on the targeted bacterium.

Even if RT-qPCR detected *cvaC-1* traces in desiccated tissues, the PCA analysis distinctly separated the samples into different clusters, based on their infection status and treatment, providing different significant insights on the efficacy of the assays herein developed.

The Spearman correlation coefficient (*r* = 0.90) indicates a strong positive correlation between the Cq values obtained with RT-qPCR for the *cvaC-1* transcript and those from the qPCR targeting the *rimM* gene (Amoia et al., 2023). This further supports that RT-qPCR assay for *cvaC-1* transcript is reliable in detecting and quantifying the presence of bacterial genetic material. The lower Cq values observed in “green-twigs” samples confirm as expected, a more elevated bacterial viability in this tissue, while higher values, such as those recorded in the “withered-twigs” and “dead-twigs” samples, indicate a reduced presence of bacterial genetic material, suggesting lower bacterial activity or the presence of non-growing bacterial cells.

A critical aspect emerging from the results concerns the impact of tetracycline HCl treatment. Treated samples show more considerable variability of Cq values compared to untreated ones, suggesting that the antibiotic treatment significantly affected bacterial cell viability. Specifically, in treated samples, the increased Cq values of RT-qPCR compared to those of conventional qPCR suggest that the antibiotic negatively impacted bacterial multiplication. Quantification cycles in “withered-twigs,” “dead-twigs” and those treated with tetracycline HCl were consistently higher in RT-qPCR than the qPCR, remarking that conventional detection approaches, targeting bacterial DNA, are not suitable to retrieve information about the viability and growing stage of the bacterium in the infected tissues, thus not suitable for example, to estimate the effect and the efficacy of anti-bacterial treatments *in planta*.

In conclusion, our work sheds light on molecular pathways in the *Xf* life cycle, both *in vitro* and *in planta*, disclosing that bacteriocins may play a critical role in the successful bacterial host colonization and to conquer the xylem niche by successfully competing with endophytes. However, it still remains unclear how these interactions may result in severe diseases or latent infections in other host-strain combinations.

On the other hand, the high level of *cvaC-1* expression *in planta* can be exploited as a diagnostic marker, either to improve the sensitivity of the current and classical diagnostic tests or to assess if the detectable bacterial DNA is linked to an active *Xf* multiplication stage of the infection or to non-growing bacterial cells unable to further support the infection process in the host plant. This represents a crucial aspect for estimating the phytosanitary risks linked to the detection of the bacterium, especially for those host plants from which it is challenging to recover active growing colonies on artificial media and make an assessment of the effect of control means on the viability of the bacterium upon the treatments. Nonetheless, the *cvaC-1*-based protocols proved to be a promising tool for monitoring the effect of the applications of antimicrobial compounds to control the bacterium in infected plants.

Previously, attempts have been made to develop molecular assays able to quantify viable cells (v-qPCR) i.e., with intact membranes, from dead ones displaying membrane damages (Sicard et al., 2019; Baró et al., 2020). However, these approaches require pretreatment with DNA-binding dyes and complex bacterial isolation protocols from the host plants, which ultimately are not suitable for routine detection. Indeed, the main v-qPCR disadvantage is the possible overestimation of cell mortality, as the exposure and photoactivation conditions, required to bind the DNA from dead cells to the dyes, could also cause damage to viable cells. The high throughput RT-qPCR assay herein developed allows for adapting the standard total nucleic extraction protocols to rapidly assess, based on the Cq values and their ratio (Cq recovered for DNA *vs* Cq yielded for the RNA transcript), if an active bacterial multiplication occurs in the infected plants or this is impaired and only non-growing bacterial cells are detected. Indeed, the two-step RT-PCR protocol herein developed, even if slightly less sensitive than the RT-qPCR test and more time-consuming, proved to selectively produce amplicon only in host tissues supporting active bacterial multiplication. RNA-based PCR assays for specific detection and quantification of viable bacterial cells have been previously developed for culturable and unculturable bacteria (Golmohammadi et al., 2012; Fujikawa et al., 2020; Wang et al., 2023), providing an efficient alternative diagnostic tool to indirectly estimate viable bacterial cells. The work herein described, while contributing to discover the highly transcribed genes of the target pathogen in the infected plants, represents the first attempt to use a RNA-based approach to improve the detection of this emergent plant pathogen upon performing several *in planta* assessments. This newly developed assay could be useful not only to evaluate the effects of the application of antimicrobial compounds to control *Xf* but also in research context, for monitoring the progression of *Xf* infection in relation to the host plant response, and for predicting the pathogen-host compatibility.

## Data Availability

The raw data supporting the conclusions of this article are included in this article/Supplementary material. Further inquiries can be directed to the corresponding authors. The sequencing dataset presented in this study was deposited in the National Center for Biotechnology Information (NCBI) BioProject repository https://www.ncbi.nlm.nih.gov under accession number PRJNA1141272.

## Footnotes

1
http://www.vertis-biotech.com/

2
https://www.bioinformatics.babraham.ac.uk/projects/fastqc/

3
https://www.ncbi.nlm.nih.gov/

4
https://www.bioinformatics.babraham.ac.uk/projects/seqmonk/
