# Supplementary figures and images for: Preliminary transcriptomic analyses reveal in vitro and in planta overexpression of various bacteriocins in Xylella fastidiosa
Source: Front Microbiol. 2025 Feb 21;16:1501741. doi: 10.3389/fmicb.2025.1501741 (PMC11885251; doi:10.3389/fmicb.2025.1501741)

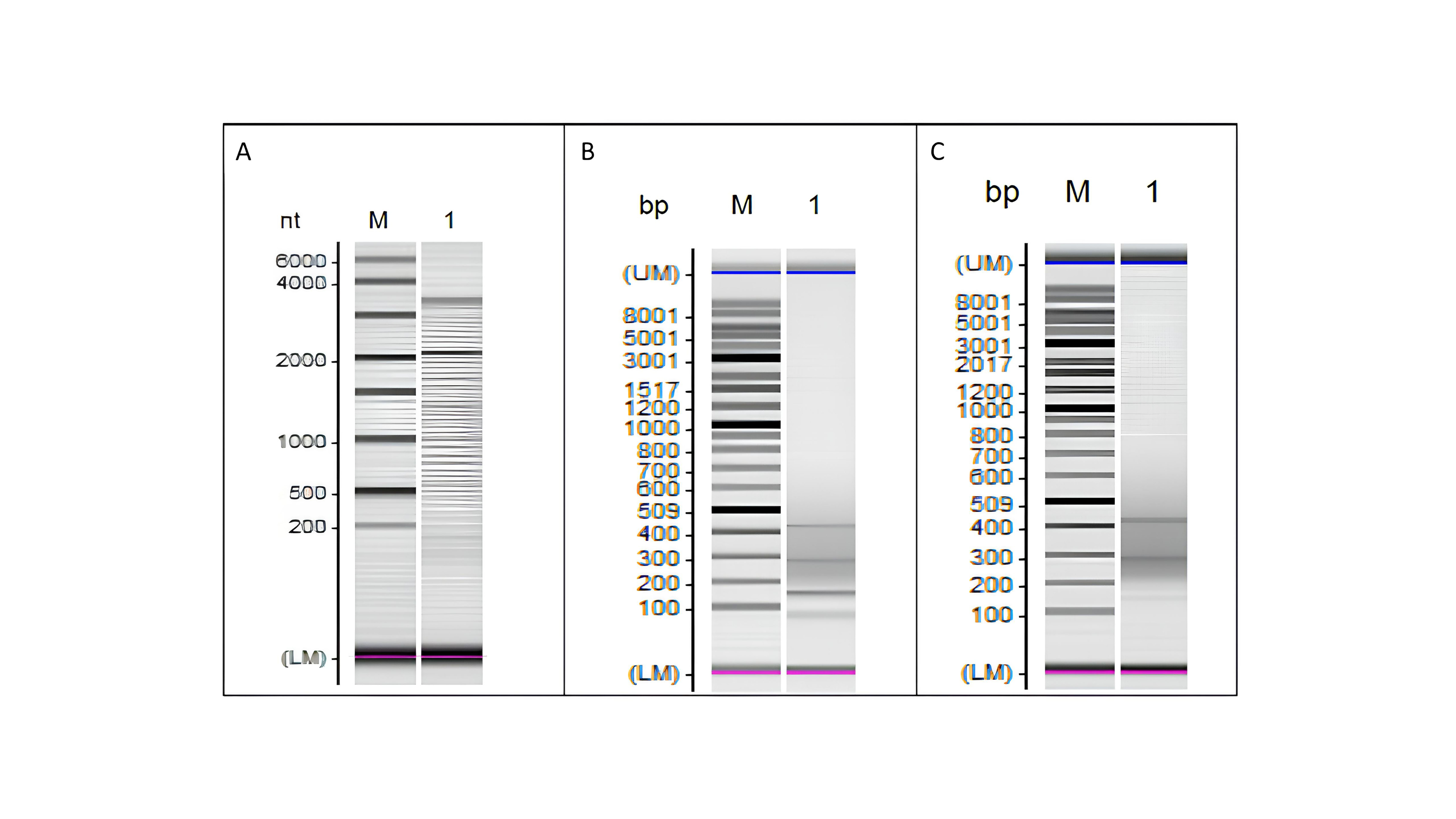

Supplement: Supplementary file 2 [file Image_1.JPEG]

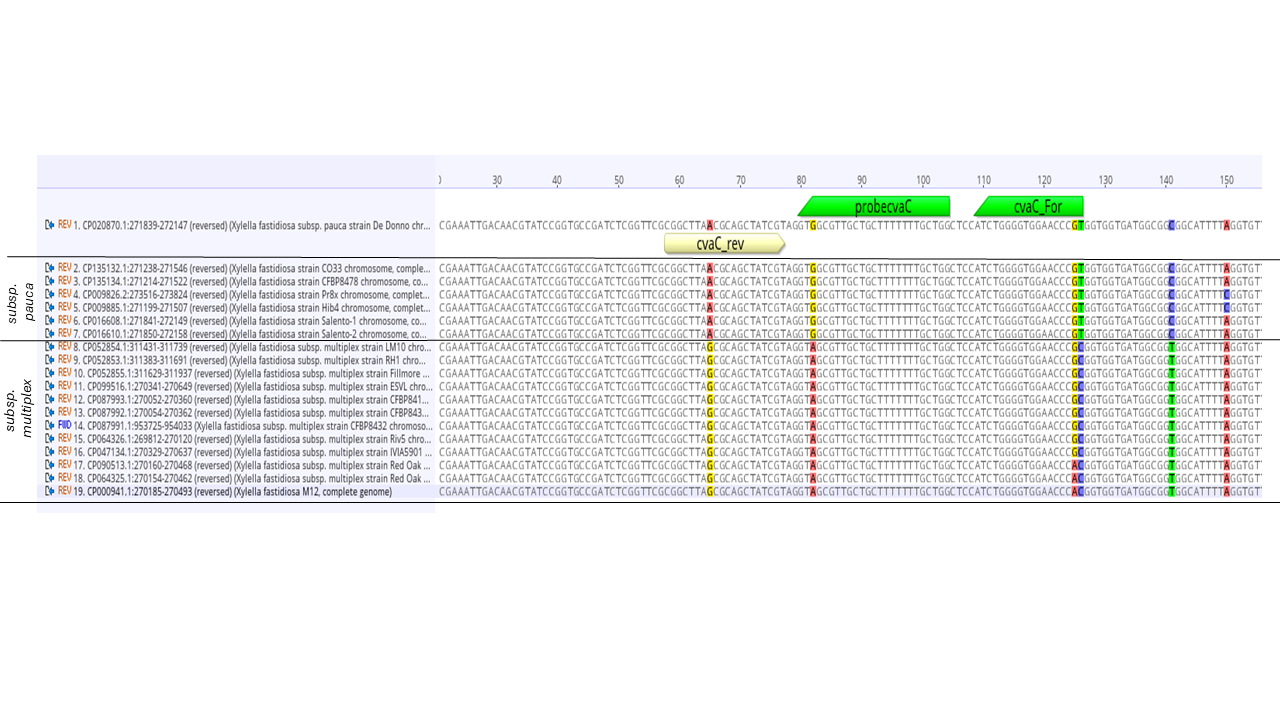

Supplement: Supplementary file 3 [file Image_2.PNG]

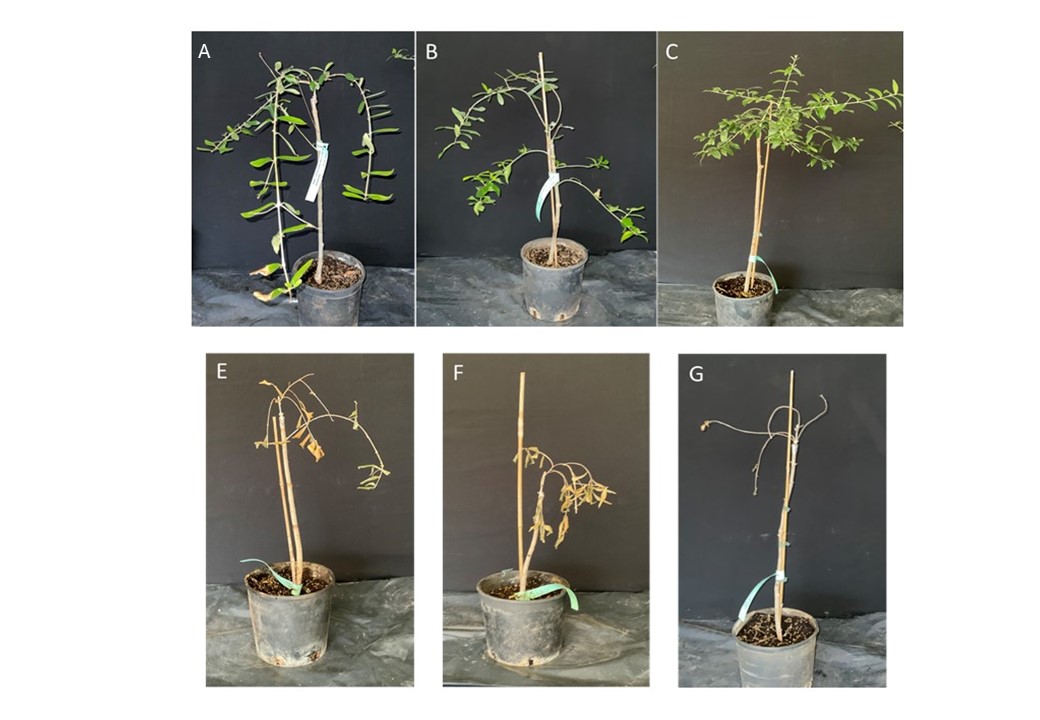

Supplement: Supplementary file 4 [file Image_3.JPEG]

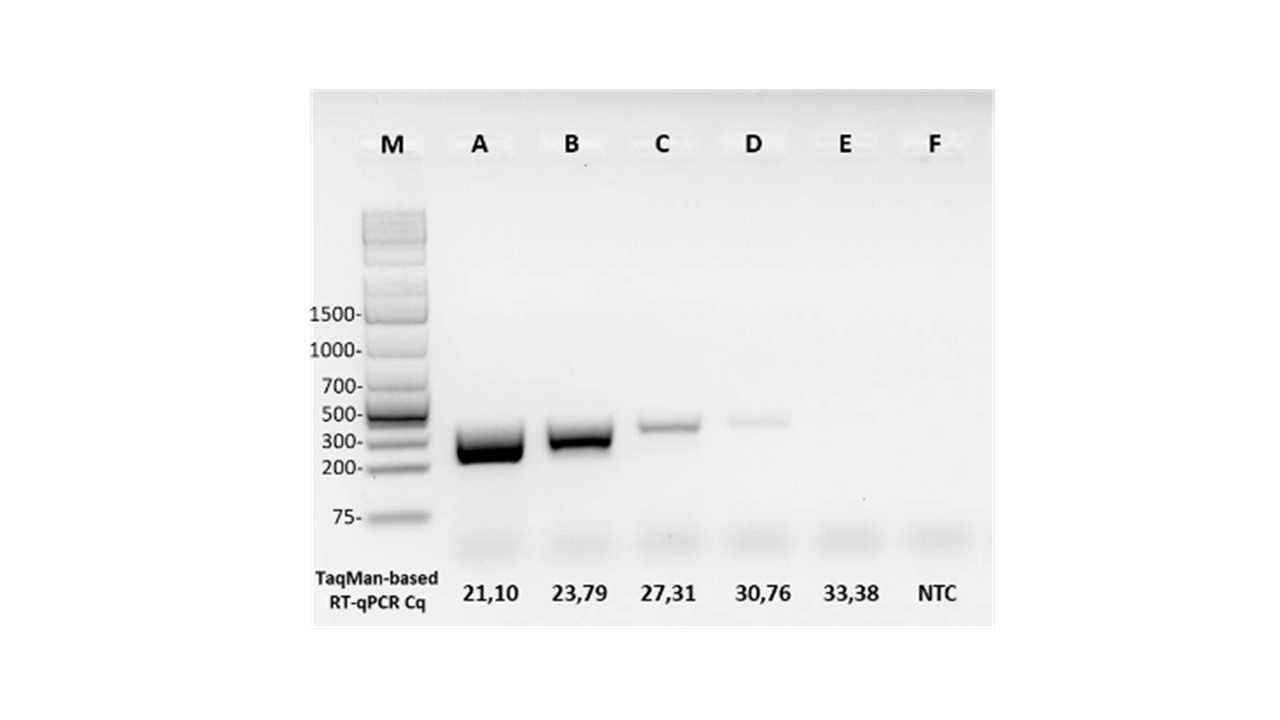

Supplement: Supplementary file 5 [file Image_4.JPEG]

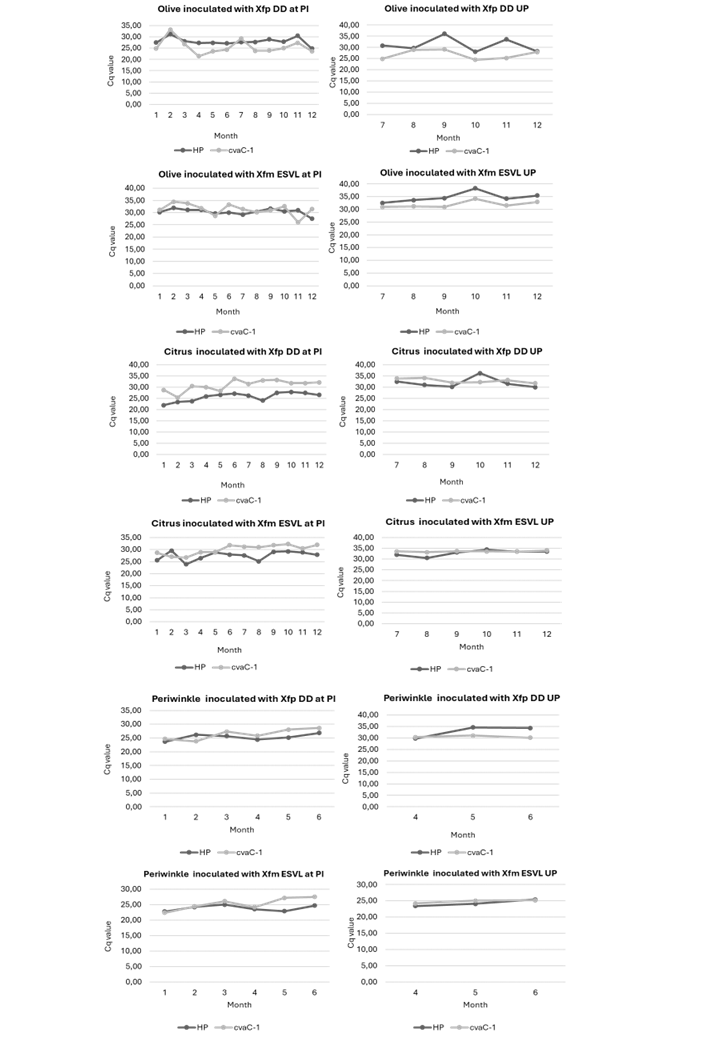

Supplement: Supplementary file 6 [file Image_5.PNG]
