# Supplementary material for: Preliminary transcriptomic analyses reveal in vitro and in planta overexpression of various bacteriocins in Xylella fastidiosa
Source: Front Microbiol. 2025 Feb 21;16:1501741. doi: 10.3389/fmicb.2025.1501741 (PMC11885251; doi:10.3389/fmicb.2025.1501741)
Supplement: Supplementary file 7 [file Table_1.DOCX]

Supplementary Material

# Supplementary Figures and Tables Captions

**Supplementary Figure 1.** Quality control of the RNA sample used in dual RNA-seq library. Analysis of the (A) total RNA sample, (B) PCR-amplified cDNA sample and (C) the size-fractionated cDNA sample on a Shimadzu MultiNA microchip electrophoresis system. M = Molecular RNA marker (a); 100 bp ladder (b,c).

**Supplementary Figure 2.** Multiple alignments of *cvaC-1* gene sequences of representative *Xf* isolates (subsp. *pauca* and *multiplex*) deposited in GenBank. The positions of the forward and reverse primers and probe, used in TaqMan-based RT-qPCR, are indicated with arrows.

Supplementary Figure 3. Representative symptomatic and non-symptomatic *Xf*-infected plants used for the evaluation of the *cvaC-1* transcript accumulation in vegetating or desiccated tissues. RT-qPCR generates an average low Cq value (18.11) for the symptomless plants (*i.e*., viable shoots; A, B, C). Conversely, for the dead plants (E, F, G) RT-qPCR gave an average Cq of 29.99, whereas RT-PCR provided a clear amplicon in the first case and did not produce any visible amplicon in the second condition.

**Supplementary Figure 4.** RT-PCR diagnostic sensitivity. Electrophoresis gel showing the RT-PCR products of five ten-fold dilutions (from 1 to 10^−5^; Lane A-E) of cDNA synthesized from RNA recovered from a *Xfp*-infected olive using the Maxwell® RSC Plant RNA kit. M: GeneRuler 1 kb Plus DNA Ladder (Thermo Scientific) ladder; Lane F: No template control (NTC). The corresponding Cq values obtained in TaqMan-based RT-qPCR assay are reported below.

**Supplementary Figure 5.** Results of qPCR (following Amoia et al., 2023 protocol) and RT-qPCR (targeting *cvaC-1* transcript) assays for olive, citrus (overall 12-months trial) and periwinkle (overall 6-months trial). Twigs with two inoculation points (PI) were analyzed each month. From the third for periwinkle and the sixth months post inoculation for olive and citrus in addition, twig portions collected at 10 cm above the last inoculation point (UP) were sampled and analyzed for the evaluation of the *Xf* progression and cell activity. The month of sampling and the quantification cycle (Cq value) are reported on the x-axis and y-axis, respectively.

**Supplementary Table 1.** Dual RNAseq from plant S2 and bacterial RNAseq data from Xfp DD, APL 64 and APL 69 genomes are reported. Columns indicate the chromosome coordinate of the identified genes, the beginning and the end nucleotide for each gene, and their protein ID description and locus tag. Raw and normalized mapped reads are reported.

**Supplementary Table 2.** List of 48 olive-infected plants used for the comparison between conventional qPCR and RT-PCR assays for *cvaC-1* transcript integrity evaluation. The status of each plant (alive, dead or wilted), Cq values of conventional qPCR and RT-qPCR targeting *cvaC-1* gene are reported. The presence (+) or absence (-) of the amplicon band in electrophoresis gel is also shown. HC: Healthy control; NA: not absorbance.

**Supplementary Table 3.** Descriptive statistics and statistical analysis of main differences between samples analyzed by conventional qPCR and RT-qPCR targeting *cvaC-1* gene.
