# Supplementary material for: Preliminary transcriptomic analyses reveal in vitro and in planta overexpression of various bacteriocins in Xylella fastidiosa
Source: Front Microbiol. 2025 Feb 21;16:1501741. doi: 10.3389/fmicb.2025.1501741 (PMC11885251; doi:10.3389/fmicb.2025.1501741)
Supplement: Supplementary file 8 [file Table_2.DOCX]

| **Supplementary Table 2.** List of 48 olive infected plants used for the comparison between conventional qPCR and RT-PCR assays for *cvaC-1* transcript integrity evaluation. The status of each plant (alive, dead or wilted), Cq values of conventional qPCR and RT-qPCR targeting *cvaC-1* gene are reported. The presence (+) or absence (-) of amplicon band in electrophoresis gel is also shown. HC: Healthy control; NA: not absorbance | | | | | |
| --- | --- | --- | --- | --- | --- |
| **ID Plant** | **Status** | **Treated/ Non-Treated** | **qPCR Harper et al., 2010** | **RT-qPCR cvaC-1** | **RT-PCR electrophoresis gel** |
| 1 | ALIVE | non treated | 21,43 | 15,96 | + |
| 2 | ALIVE | non treated | 20,51 | 16,24 | + |
| 3 | WILTED | non treated | 25,34 | 31,62 | - |
| 4 | ALIVE | non treated | 21,24 | 16,38 | + |
| 5 | ALIVE | non treated | 20,55 | 15,86 | + |
| 6 | ALIVE | non treated | 21,17 | 16,27 | + |
| 7 | ALIVE | non treated | 21,40 | 17,51 | + |
| 8 | WILTED | non treated | 23,94 | 30,67 | - |
| 10 | WILTED | non treated | 25,03 | 30,26 | - |
| 11 | DEAD | non treated | 26,39 | 29,70 | - |
| 13 | WILTED | non treated | 25,04 | 30,60 | - |
| 15 | ALIVE | non treated | 19,25 | 14,47 | + |
| 16 | ALIVE | non treated | 20,83 | 16,21 | + |
| 17 | ALIVE | non treated | 20,57 | 15,76 | + |
| 19 | DEAD | non treated | 28,77 | 31,80 | - |
| 22 | DEAD | non treated | 25,46 | 30,49 | - |
| 24 | DEAD | non treated | 24,90 | 31,60 | - |
| 25 | DEAD | non treated | 24,33 | 30,32 | - |
| 27 | WILTED | non treated | 25,49 | 27,24 | - |
| 28 | ALIVE | non treated | 20,63 | 17,04 | + |
| 30 | DEAD | non treated | 25,26 | 31,06 | - |
| 31 | WILTED | non treated | 22,43 | 28,14 | - |
| 32 | DEAD | non treated | 23,38 | 27,49 | - |
| 34 | WILTED | non treated | 24,37 | 29,28 | - |
| 35 | ALIVE | non treated | 20,09 | 17,16 | + |
| 36 | ALIVE | non treated | 19,80 | 16,03 | + |
| 37 | DEAD | non treated | 26,64 | 30,35 | - |
| 38 | ALIVE | non treated | 20,68 | 18,31 | + |
| 39 | DEAD | non treated | 26,02 | 28,65 | - |
| 40 | ALIVE | non treated | 19,89 | 16,99 | + |
| 41 | DEAD | non treated | 25,90 | 30,51 | - |
| 42 | DEAD | non treated | 25,96 | 28,47 | - |
| 49 | ALIVE | non treated | 23,21 | 19,63 | + |
| 51 | ALIVE | non treated | 21,09 | 17,01 | + |
| 52 | DEAD | non treated | 23,79 | 27,89 | - |
| 54 | ALIVE | non treated | 19,59 | 16,23 | + |
| 44 | ALIVE | treated | 26,16 | 30,98 | - |
| 46 | ALIVE | treated | 25,73 | 27,12 | - |
| 43 | ALIVE | treated | 21,42 | 18,23 | + |
| 45 | ALIVE | treated | 23,13 | 19,54 | + |
| 47 | ALIVE | treated | 23,00 | 19,39 | + |
| 48 | DEAD | treated | 30,56 | 31,87 | - |
| HC-1 | ALIVE |  | NA | NA | - |
| HC-2 | ALIVE |  | 34,31 | 31,56 | - |
| HC-3 | ALIVE |  | NA | 32,37 | - |
| HC-4 | ALIVE |  | 33,91 | 32,47 | - |
| HC-5 | ALIVE |  | NA | 31,20 | - |
| HC-6 | ALIVE |  | NA | 32,58 | - |
