# Supplementary material for: Preliminary transcriptomic analyses reveal in vitro and in planta overexpression of various bacteriocins in Xylella fastidiosa
Source: Front Microbiol. 2025 Feb 21;16:1501741. doi: 10.3389/fmicb.2025.1501741 (PMC11885251; doi:10.3389/fmicb.2025.1501741)
Supplement: Supplementary file 9 [file Table_3.DOCX]

| **Supplementary Table 3.** Descriptive statistics and statistical analysis of main differences between samples analyzed by conventional qPCR and RT-qPCR targeting *cvaC-1* gene. | | | | | | | | |
| --- | --- | --- | --- | --- | --- | --- | --- | --- |
|  | **Treated** | | **Green-twigs** | | **Dead_withered-twigs** | | **Healthy control** | |
|  | qPCR Harper et al., 2010 | RT-qPCR cvaC-1 | qPCR Harper et al., 2010 | RT-qPCR cvaC-1 | qPCR Harper et al., 2010 | RT-qPCR cvaC-1 | qPCR Harper et al., 2010 | RT-qPCR cvaC-1 |
| Mean | 25 | 24.52 | 20.7 | 16.65 | 25.18 | 29.8 | 34.7 | 32.53 |
| Std. Deviation | 3.26 | 6.22 | 0.91 | 1.14 | 1.38 | 1.43 | 0.48 | 1.33 |
| Std. Error of Mean | 1.33 | 2.54 | 0.22 | 0.28 | 0.32 | 0.33 | 0.19 | 0.54 |
| Coefficient of variation | 13.04% | 25.35% | 4.40% | 6.85% | 5.48% | 4.81% | 1.37% | 4.08% |
| Multiple comparison (Kruskal-Wallis test) | abcd | abcd | ab | a | bc | cd | d | cd |
